# Management and Outcomes Following Surgery for Gastrointestinal Typhoid: An International, Prospective, Multicentre Cohort Study

**DOI:** 10.1007/s00268-018-4624-8

**Published:** 2018-05-03

**Authors:** GlobalSurg Collaborative, Theophilus T. K. Anyomih, Thomas M. Drake, James Glasbey, J. Edward Fitzgerald, Riinu Ots, Ewen M. Harrison, Stephen Tabiri, Aneel Bhangu, Adesoji O. Ademuyiwa, Maria Lorena Aguilera, Philip Alexander, Sara W. Al-Saqqa, Giuliano Borda-Luque, Ainhoa Costas-Chavarri, Thomas M. Drake, Faustin Ntirenganya, J. Edward Fitzgerald, Stuart J. Fergusson, James Glasbey, J. C. Allen Ingabire, Lawani Ismaïl, Hosni Khairy Salem, Anyomih Theophilus Teddy Kojo, Marie Carmela Lapitan, Richard Lilford, Andre L. Mihaljevic, Dion Morton, Alphonse Zeta Mutabazi, Dmitri Nepogodiev, Adewale O. Adisa, Riinu Ots, Francesco Pata, Thomas Pinkney, Tomas Poškus, Ahmad Uzair Qureshi, Antonio Ramos-De la Medina, Sarah Rayne, Catherine A. Shaw, Sebastian Shu, Richard Spence, Neil Smart, Stephen Tabiri, Ewen M. Harrison, A. O. Ademuyiwa, S. Tabiri, Cutting Edge Manipal, M. Mohan, J. Jeyakumar, Ashrarur Rahman Mitul, Khalid Mahmud, Margub Hussain, Hafiz Hakim, Tapan Kumar, Antje Oosterkamp, Francis Abantanga, Kwaku Boakye-Yiadom, Mohammed Bukari, Frank Owusu, Joseph Awuku-Asabre, Stephen Tabiri, Lemuel Davies Bray, S. S. Prasad, Anand Kirishnan, Nidhi Gyanchandani, Bylapudi Seshu Kumar, Muthukumaran Rangarajan, Sriram Bhat, Anjana Sreedharan, S. V. Kinnera, Yella Reddy, Caranj Venugopal, Sunil Kumar, Abhishek Mittal, Shravan Nadkarni, Harish Neelamraju Lakshmi, Puneet Malik, Neel Limaye, Srinivas Pai, Pratik Jain, Monty Khajanchi, Savni Satoskar, Rajeev Satoskar, Abid Bin Mahamood, Ross Coomber, Kenneth Johnson, Jennifer Nowers, Aminu Mohammad, Lofty-John Anyanwu, Abdulrahman Sheshe, Alaba Adesina, Olubukola Faturoti, Ogechukwu Taiwo, Muhammad Habib Ibrahim, Abdulrasheed A. Nasir, Siyaka Itopa Suleiman, Adewale Adeniyi, Opeoluwa Adesanya, Ademola Adebanjo, Omolara Williams, Kazeem Atobatele, Ayokunle Ogunyemi, Mobolaji Oludara, Olabode Oshodi, Roland Osuoji, Adesoji Ademuyiwa, AbdulRazzaq Oluwagbemiga Lawal, Felix Alakaloko, Olumide Elebute, Adedapo Osinowo, Christopher Bode, Abidemi Adesuyi, Adesoji Tade, Adeleke Adekoya, Collins Nwokoro, Omobolaji O. Ayandipo, Taiwo Akeem Lawal, Akinlabi E. Ajao, Samuel Sani Ali, Babatunde Odeyemi, Samson Olori, Ademola Popoola, Ademola Adeyeye, James Adeniran, Kamran Faisal Bhopal, Zainab Iftikhar, Muhammad Mohsin Furqan, Bakhtiar Nighat, Masood Jawaid, Abdul Khalique, Ahsan Zil-E-Ali, Anam Rashid, Hasnain Abbas Dharamshi, Tahira Naqvi, Ahmad Faraz, Abdul Wahid Anwar, Tahir Muhammad Yaseen, Ghina Shamim Shamsi, Ghina Shamsi, Tahir Yaseen, Wahid Anwer, Prasad Pitigala Arachchi, Wanigasekara Senanayake Mudiyanselage Kithsiri Janakantha Senanayake, Lalith Asanka Jayasooriya Jayasooriya Arachchige, Sivasuriya Sivaganesh, Dulan Irusha Samaraweera, Vimalakanthan Thanusan, Lawani Ismaïl, Stephen Tabiri, Yong Yong Tew, Adesoji O. Ademuyiwa, Tanzeela Gala, Fernande Djivoh, Lawani Ismaïl, Francis Dossou, Djifid Morel Seto, Dansou Gaspard Gbessi, Bruno Noukpozounkou, Yacoubou Imorou Souaibou, Kpèmahouton René Keke, Fred Hodonou, Ernest Yemalin Stephane Ahounou, Thierry Alihonou, Max Dénakpo, Germain Ahlonsou, Stephen Tabiri, Anyomih Theophilus Teddy Kojo, Dickson Bandoh, Francis Abantanga, Martin Kyereh, Hamza Asumah, Eric Kofi Appiah, Paul Wondoh, Adam Gyedu, Charles Dally, Kwabena Agbedinu, Michael Amoah, Abiboye Yifieyeh, Frank Owusu, Mabel Amoako-Boateng, Makafui Dayie, Richmond Hagan, Sam Debrah, Micheal Ohene-Yeboah, Joe-Nat Clegg-Lampety, Victor Etwire, Jonathan Dakubo, Samuel Essoun, William Bonney, Hope Glover-Addy, Samuel Osei-Nketiah, Joachim Amoako, Niiarmah Adu-Aryee, William Appeadu-Mensah, Antoinette Bediako-Bowan, Florence Dedey, Mattew Ekow, Emmanuel Akatibo, Musah Yakubu, Hope Edem Kofi Kordorwu, Kwasi Asare-Bediako, Enoch Tackie, Kenneth Aaniana, Emmanuel Acquah, Richard Opoku-Agyeman, Anthony Avoka, Kwasi Kusi, Kwame Maison, Frank Enoch Gyamfi, Gandau Naa Barnabas, Saiba Abdul-Latif, Philip Taah Amoako, Anthony Davor, Victor Dassah, Enoch Dagoe, Prince Kwakyeafriyie, Elliot Akoto, Eric Ackom, Ekow Mensah, Ebenezer Takyi Atkins, Christian Lari Coompson, Taha Yusufali, Hussein Mohammed, Justus Lando, Robert Parker, Wairimu Ndegwa, Feng Yih Chai, Siti Mohd Desa Asilah, Khuzaimah Zahid Syibrah, Pui Xin Chin, Afizah Salleh, Nur Zulaika Riswan, April Camilla Roslani, Hoong-Yin Chong, Nora Abdul Aziz, Keat-Seong Poh, Chu-Ann Chai, Sandip Kumar, Mustafa Mohammed Taher, Nik Ritza Kosai, Dayang Nita Abdul Aziz, Reynu Rajan, Rokayah Julaihi, Durvesh Lacthman Jethwani, Muhammad Taqiyuddin Yahaya, Nik Azim Nik Abdullah, Susan Wndy Mathew, Kuet Jun Chung, Milaksh Kumar Nirumal, R. Goh Ern Tze, Syed Abdul Wahhab Eusoffee Wan Ali, Yiing Yee Gan, Jesse Ron Swire Ting, Samuel S. Y. Sii, Kean Leong Koay, Yi Koon Tan, Alvin Ee Zhiun Cheah, Chui Yee Wong, Tuan Nur’Azmah Tuan Mat, Crystal Yern Nee Chow, Prisca A. L. Har, Yishan Der, Yong Yong Tew, Fitjerald Henry, Xinwei Low, Ya Theng Neo, Hian Ee Heng, Shu Ning Kong, Cheewei Gan, Yi Ting Mok, Yee Wen Tan, Kandasami Palayan, Mahadevan Deva Tata, Yih Jeng Cheong, Kuhaendran Gunaseelan, Wan Nurul ‘Ain Wan Mohd Nasir, Pigeneswaren Yoganathan, Eu Xian Lee, Jian Er Saw, Li Jing Yeang, Pei Ying Koh, Shyang Yee Lim, Shuang Yi Teo, Akinlabi Ajao, Omobolaji Ayandipo, Taiwo Lawal, Abdussemiu Abdurrazzaaq, Muslimat Alada, Abdulrasheed Nasir, James Adeniran, Olufemi Habeeb, Ademola Popoola, Ademola Adeyeye, Ademola Adebanjo, Opeoluwa Adesanya, Adewale Adeniyi, Henry Mendel, Bashir Bello, Umar Muktar, Adedapo Osinowo, Thomas Olagboyega Olajide, Oyindamola Oshati, George Ihediwa, Babajide Adenekan, Victor Nwinee, Felix Alakaloko, Adesoji Ademuyiwa, Olumide Elebute, Abdulrazzaq Lawal, Chris Bode, Mojolaoluwa Olugbemi, Alaba Adesina, Olubukola Faturoti, Oluwatomi Odutola, Oluwaseyi Adebola, Clement Onuoha, Ogechukwu Taiwo, Omolara Williams, Fatai Balogun, Olalekan Ajai, Mobolaji Oludara, Iloba Njokanma, Roland Osuoji, Stephen Kache, Jonathan Ajah, Jerry Makama, Ahmed Adamu, Suleiman Baba, Mohammad Aliyu, Shamsudeen Aliyu, Yahaya Ukwenya, Halima Aliyu, Tunde Sholadoye, Muhammad Daniyan, Oluseyi Ogunsua, Lofty-John Anyanwu, Abdurrahaman Sheshe, Aminu Mohammad, Samson Olori, Philip Mshelbwala, Babatunde Odeyemi, Garba Samson, Oyediran Kehinde Timothy, Sani Ali Samuel, Anthony Ajiboye, Ademola Adeyeye, Isaac Amole, Olajide Abiola, Akin Olaolorun, Najwa Nadeem, Muhammad Saqlain, Jibran Abbasy, Abdul Rehman Alvi, Tanzeela Gala, Noman Shahzad, Kamran Faisal Bhopal, Zainab Iftikhar, Muhammad Talha Butt, Syed Asaat ul Razi, Asdaq Ahmed, Ali Khan Niazi, Ibrahim Raza, Fatima Baluch, Ahmed Raza, Ahmad Bani-Sadar, Ahmad Uzair Qureshi, Muhammad Adil, Awais Raza, Mahnoor Javaid, Muhammad Waqar, Maryam Ali Khan, Mohammad Mohsin Arshad, Mohammadasim Amjad, J. C. Allen Ingabire, Alphonse Zeta Mutabazi, Norbert Uzabumwana, Dieudonne Duhoranenayo, Joe-Nat Clegg-Lamptey, Osman Imoro, Owusu Emmanuel Abem, Paul Wondoh, Danjuma Sale, Lawal Abdullahi, Olabisi Osagie, Omolara Faboya, Adedeji Fatuga, Agboola Taiwo, Emeka Nwabuoku, Zain Ali Khan, Jennifer Rickard, Choy Ling Tan, Jia Yng Siaw, Sir Young Yam, Ling Wilson, Mohamed Rezal Abdul Aziz

**Affiliations:** Department of Surgery, University for Development Studies, School of Medicine and Health Sciences and Tamale Teaching Hospital, Tamale, Ghana

## Abstract

**Background:**

Gastrointestinal perforation is the most serious complication of typhoid fever, with a high disease burden in low-income countries. Reliable, prospective, contemporary surgical outcome data are scarce in these settings. This study aimed to investigate surgical outcomes following surgery for intestinal typhoid.

**Methods:**

Two multicentre, international prospective cohort studies of consecutive patients undergoing surgery for gastrointestinal typhoid perforation were conducted. Outcomes were measured at 30 days and included mortality, surgical site infection, organ space infection and reintervention rate. Multilevel logistic regression models were used to adjust for clinically plausible explanatory variables. Effect estimates are expressed as odds ratios (ORs) alongside their corresponding 95% confidence intervals.

**Results:**

A total of 88 patients across the GlobalSurg 1 and GlobalSurg 2 studies were included, from 11 countries. Children comprised 38.6% (34/88) of included patients. Most patients (87/88) had intestinal perforation. The 30-day mortality rate was 9.1% (8/88), which was higher in children (14.7 vs. 5.6%). Surgical site infection was common, at 67.0% (59/88). Organ site infection was common, with 10.2% of patients affected. An ASA grade of III and above was a strong predictor of 30-day post-operative mortality, at the univariable level and following adjustment for explanatory variables (OR 15.82, 95% CI 1.53–163.57, *p* = 0.021).

**Conclusions:**

With high mortality and complication rates, outcomes from surgery for intestinal typhoid remain poor. Future studies in this area should focus on sustainable interventions which can reduce perioperative morbidity. At a policy level, improving these outcomes will require both surgical and public health system advances.

**Electronic supplementary material:**

The online version of this article (10.1007/s00268-018-4624-8) contains supplementary material, which is available to authorized users.

## Introduction

Typhoid fever is caused primarily by a gram-negative *Salmonella enterica* species *(Salmonella typhi* and *Salmonella paratyphi)*. It is commonly transmitted via the faeco-oral route and is epidemic in areas with poor sanitation and limited availability of clean water. One of the common complications of typhoid fever is gastrointestinal perforation, which usually requires emergency surgery [1]. These perforations normally occur in the jejunum or ileum, but there have been several reported cases of colonic and even gallbladder perforations [2]. Perforation typically occurs 2–3 weeks after onset of the disease [3]. The incidence and outcomes following emergency surgery for typhoid perforation are poorly described with existing studies either single centre or retrospective in nature [4].

The disease is rare in countries with good sanitation, but has continued to be a public health concern in many low- and middle-income countries (LMICs) [5]. The high morbidity and mortality associated with intestinal typhoid is exacerbated by lack of access to medical facilities in remote and rural areas, where delays in presentation can lead to perforation and severe complications in both children and adults [6]. The reported mortality rates in LMICs can be as high as 62–80% and are highest where presentation to medical services is delayed [7, 8]. In survivors of intestinal perforation, there is a high level of morbidity, exerting considerable societal, economic and healthcare burdens.

Difficulties in the prompt diagnosis of typhoid fever serve to compound these issues. The gold standard test for diagnosis of enteric fever is bone marrow culture [9]. Blood cultures to detect *S. Typhi* and *S. Paratyphi* are possible, but have a lower sensitivity than bone marrow sampling. Unfortunately, in most LMICs where the disease is endemic, both blood and bone marrow culture are unavailable, unaffordable or not routinely performed [10].

The GlobalSurg Collaborative has demonstrated the feasibility of conducting international high-quality data collection into low-resource centres. This study aimed to investigate surgical outcomes following surgery for gastrointestinal typhoid globally, using an international collaborative research network.

## Methods

### Study settings

Two international, multicentre, prospective, observational cohort studies were conducted according to pre-specified, published protocols: GlobalSurg 1 between 1 July 2014 and 31 December 2014, and GlobalSurg 2 between 1 January 2016 and 31 July 2016 (NCT02662231 and NCT02179112) [11, 12]. The GlobalSurg group collaborative network methodology has been previously described in detail elsewhere [13]. Local investigators were responsible for ethics or audit registration according to regional or institutional governance protocols. This study is reported according to the Strengthening the Reporting of Observational Studies in Epidemiology (STROBE) guidelines [14].

### Patients and procedures

This study is a pre-specified, subgroup analysis of patients who underwent emergency abdominal surgery for typhoid-associated gastrointestinal disease with perforation (including gallbladder). Any hospital providing acute surgical care around the world was eligible to enter patient data. Consecutive patients undergoing emergency (GlobalSurg 1) and emergency or elective (GlobalSurg 2) abdominal surgery were eligible for inclusion. Only patients with intra-operatively, pathologically or microbiologically confirmed typhoid infection were included within this analysis. Patients of any age undergoing surgery by any operative approach (open, laparoscopic and laparoscopic-converted procedures) were eligible for inclusion. Emergency surgery was defined as any unplanned (non-elective) operation, including reoperation after a previous procedure.

### Data collection and management

Data were collected on internationally relevant patient, disease and outcome data. This approach aimed to maximise case record completion and data accuracy. Patients were followed up for 30 days after surgery, with the day of surgery taken as day zero [11, 12]. Records were uploaded by local investigators to a secure online website provided using the Research Electronic Data Capture (REDCap) system [15]. The lead investigator at each site was responsible for data accuracy of all cases prior to data submission. Only data with high levels of completeness (> 95%) were accepted for analysis.

Patient variables included demographic details (age, gender, country, diabetes status, smoking status, American Society of Anesthesiologists (ASA) grade), operative details (time to operation, operative approach, WHO safer surgery checklist use, primary procedure performed) and outcomes (30-day mortality, 30-day reintervention, 30-day surgical site infection (SSI) and 30-day organ site infection (OSI) rates). Antibiotic prophylaxis was defined as antibiotics which were administered over the perioperative period, either at induction or during operation prior to opening a contaminated space, or antibiotic therapy that was commenced prior to operation but continued over the perioperative period. Due to the lack of microbiological data and resistance data, it was not feasible to collect data on which antibiotics were administered. As typhoid commonly affects children in low- and middle-income countries and paediatric surgery is commonly performed in separate units, we chose to compare outcomes across children and adults to provide further detail in these groups.

### Outcome variables

The primary outcome measure was the 30-day post-operative mortality rate. The secondary outcome measures included the 30-day reintervention rate, the 30-day organ space infection rate and the surgical site infection rate, defined according to the US Centre for Disease Control and Prevention (CDC) definitions for SSI [16].

SSI was defined as one of:

- Purulent drainage from the superficial or deep (fascia or muscle) incision but not from within the organ/space component of the surgical site;
- At least one of: pain or tenderness; localised swelling; redness; heat; fever; and the incision is opened deliberately or spontaneously dehisces;
- Abscess within the wound (clinically or radiologically detected).

Organ space infections were recorded separately and defined as intra-abdominal/pelvic infections detected clinically/symptomatically, radiologically or intra-operatively. Online training modules were completed by collaborators prior to data collection.

### Statistical analysis

Data were summarised using simple percentages for categorical variables, or means for continuous data which had a parametric distribution. Summary statistical tests for differences across treatment groups were performed, with the Chi-square test for categorical data and Kruskal–Wallis for continuous data. Patient groups were cross-tabulated based on age, with children being classed as under 16 years of age and adults 16 and over. Univariable models were constructed to estimate the effect size of clinically plausible explanatory variables on 30-day outcomes following surgery for typhoid perforation. Multilevel models were then used to create models adjusted for clinically plausible explanatory variables. These multilevel models adjusted for the individual patient-level risk (level 1) and then for country effects (level 2). Effect estimates are presented as odds ratios, alongside the corresponding 95% confidence intervals. Statistical significance was taken at the level of *p* < 0.05. All analyses were performed in the R statistical programming program (R Foundation for Statistical Computing, Vienna, AUT) using the Summarizer and Tidyverse packages.

## Results

**Fig. 1:**
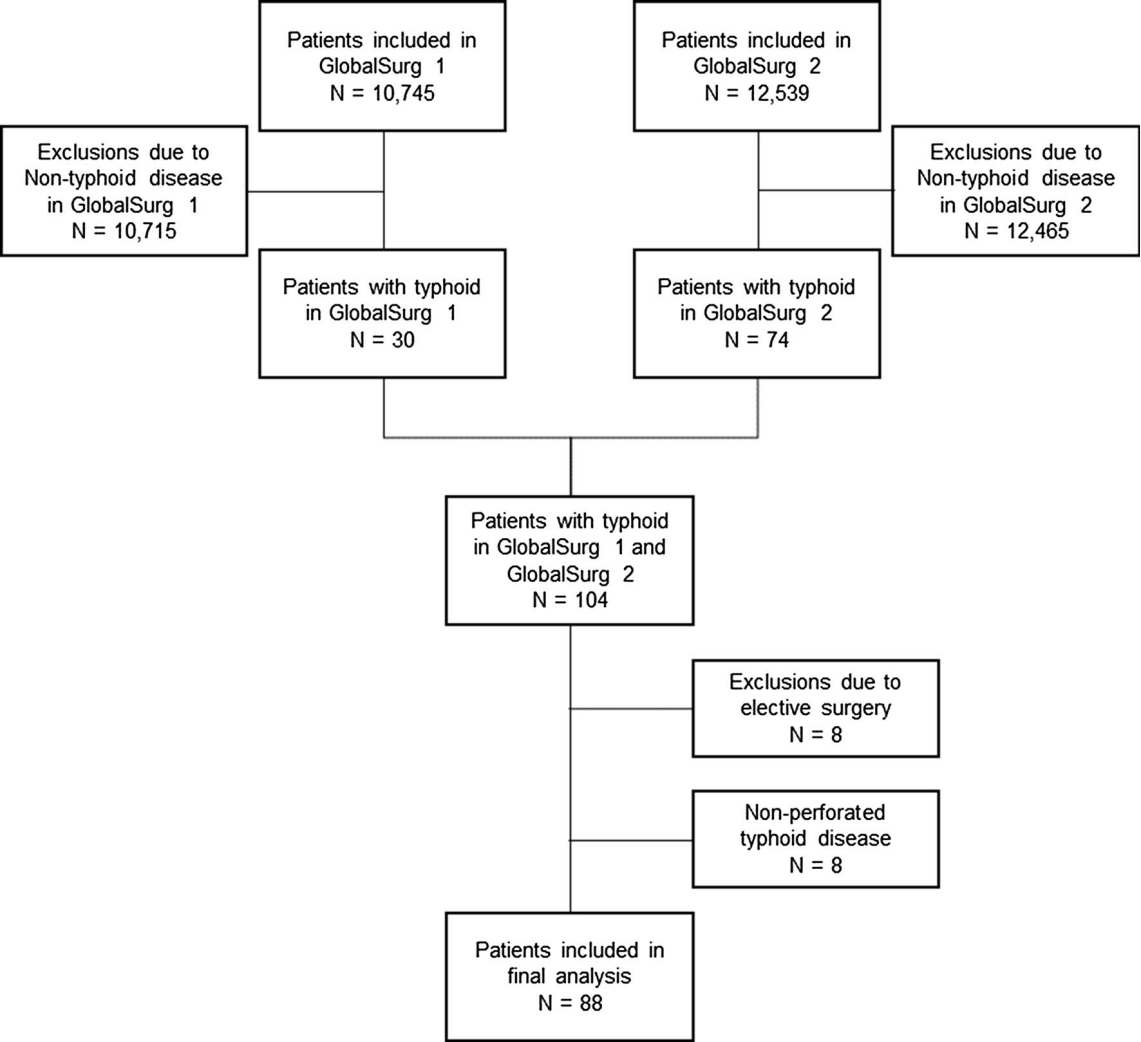
Study inclusion flowchart

A total of 88 patients from 11 countries underwent a surgical procedure for gastrointestinal typhoid perforation during the data collection periods (Fig. 1). Table 1 describes the baseline demographics of included patients. One patient was included from a high HDI country, two from middle HDI countries and 85 patients from low HDI countries. Validation performed in the GlobalSurg 2 data set demonstrated this approach to have a 93.3% case ascertainment rate and a high accuracy for categorical predictors (Cohen’s kappa coefficients > 0.90), continuous predictors (Pearson’s correlation coefficient 0.99) and mortality rate (kappa 0.91).

**Table 1:** Patient characteristics

		Child (<16 years) *N* = 34	Adult (≥16 years) *N* = 54	*p* value
Age (years)	Mean (SD)	8.2 (3.8)	31.5 (16.1)	< 0.001
Gender	Male	15 (44.1)	37 (68.5)	0.064
	Female	15 (44.1)	12 (22.2)	
	Missing	4 (11.8)	5 (9.3)	
ASA	I (Normal/healthy)	9 (26.5)	19 (35.2)	0.257
	II (Mild systemic disease)	8 (23.5)	18 (33.3)	
	III (Severe systemic disease)	12 (35.3)	11 (20.4)	
	IV (Severe systemic disease, constant threat to life)	2 (5.9)	5 (9.3)	
	V (Not expected to survive without the operation)	1 (2.9)	1 (1.9)	
	Unknown	2 (5.9)	0 (0.0)	
Smoking status	Non-smoker	34 (100.0)	40 (74.1)	0.005
	Current smoker	0 (0.0)	12 (22.2)	
	Missing	0 (0.0)	2 (3.7)	
Diabetes	No	34 (100.0)	51 (94.4)	0.162
	Yes	0 (0.0)	3 (5.6)	
Antibiotic Prophylaxis	No	3 (8.8)	5 (9.3)	0.945
	Yes	31 (91.2)	49 (90.7)	
Time to operation	<6 h	4 (11.8)	22 (40.7)	0.008
	>6 h	29 (85.3)	32 (59.3)	
	Missing	1 (2.9)	0 (0.0)	
Bowel resection	No	9 (26.5)	13 (24.1)	0.800
	Yes	25 (73.5)	41 (75.9)	
WHO safer surgery checklist used?	No, not available	12 (35.3)	10 (18.5)	0.205
	No, but available	5 (14.7)	9 (16.7)	
	Yes	17 (50.0)	35 (64.8)	

Numbers are *n* (%), unless otherwise indicated. All tests are Chi-square, where a Kruskal–Wallis test has been applied

### Patient and intervention characteristics

Of the included study population, 54 were over or equal to 16 years of age and 34 were children. The mean age of adult patients was 31.5 years (SD 16.1) and was predominantly male (68.5 vs. 22.2%). There were a considerable number of children who underwent surgery for gastrointestinal typhoid perforation (*N* = 34, 38.6%), with a mean age of 8.2 years (SD 3.8) with equal numbers of males and females (44.1 vs. 44.1%). Patients were largely healthy prior to surgery, with minimal comorbidity, and low ASA grades (61.4% ASA 1–2). Most patients were non-smokers (*N* = 74, 84.1%), and diabetes was rare in this patient cohort (*N* = 3 3.4%). The majority (*N* = 61, 69.3%) of patients were operated upon after 6 h of admission. All patients underwent open surgery (laparotomy). Sixteen (18.2%) patients had bowel resection, whilst 55 (62.5%) were oversewn or closed primarily without resection. The WHO surgical safety checklist was used in 52 (59.1%) patients. Patient characteristics are detailed in Table 1 and operative characteristics in Table 2.

**Table 2:** Operative characteristics

	Child (<16 years) *N* = 34	Adult (≥16 years) *N* = 54	*p* value
G58 Small bowel: excision of small bowel	5 (14.7)	9 (16.7)	0.272
G67 Small bowel: other open operations on small bowel	0 (0.0)	2 (3.7)	
G74 Small bowel: formation of ileostomy	2 (5.9)	10 (18.5)	
G784 Small bowel: closure of perforation	25 (73.5)	30 (55.6)	
H06 Colon: extended excision of right hemicolon	1 (2.9)	0 (0.0)	
H07 Colon: excision of right hemicolon	1 (2.9)	0 (0.0)	
H15 Colon: formation of any colonic stoma	0 (0.0)	1 (1.9)	
J23 Gallbladder: other open operations on gall bladder	0 (0.0)	1 (1.9)	
T28 Abdomen: repair of anterior abdominal wall	0 (0.0)	1 (1.9)	

Numbers are *n* (%), unless otherwise indicated. All tests are Chi-square

### Outcomes following surgery for intestinal typhoid

Outcomes following surgical intervention for typhoid are detailed in Tables 3, 4 and 5. Out of 88 patients, eight (9.1%) died within 30 days of surgery. Children had a higher mortality rate than adults (14.7 vs. 5.6%), which was not statistically significant (*p* = 0.146). At the univariable level, an ASA grade of III or above was significantly associated with 30-day post-operative mortality (OR 15.40, 95% CI 2.55–296.00, *p* = 0.013). Use of the WHO surgical checklist showed a weak association with lower 30-day mortality compared to whether a checklist was unavailable or was available but was not used (OR 0.28, 95% CI 0.05–1.36, *p* = 0.112). In the adjusted multilevel model, gender could not be included as it prevented model convergence, due to a low event rate in the respective gender groups. The association between higher mortality and ASA grade of III or above persisted in the multilevel model (OR 15.82, 95% CI 1.53–163.57, *p* = 0.021).

**Table 3:** 30-day outcomes

		Child (<16 years) *N* = 34	Adult (≥16 years) *N* = 54	*p* value
Mortality (30 days)	Alive	29 (85.3)	51 (94.4)	0.146
	Died	5 (14.7)	3 (5.6)	
Reintervention (30 days)	No	25 (73.5)	44 (81.5)	0.377
	Yes	9 (26.5)	10 (18.5)	
Surgical site infection (30 days)	No	11 (32.4)	18 (33.3)	0.924
	Yes	23 (67.6)	36 (66.7)	
Organ space infection (30 days)	No	30 (88.2)	48 (88.9)	0.684
	Yes	4 (11.8)	5 (9.3)	
	Missing	0 (0.0)	1 (1.9)	

Numbers are *n* (%), unless otherwise indicated. All tests are Chi-square

**Table 4:** Model for 30-day mortality

		Alive	Died	OR (univariable)	OR (multilevel)
Age (years)	Mean (SD)	22 (15.2)	27.6 (32)	1.02 (0.98–1.05, *p* = 0.378)	1.02 (0.98–1.06, *p* = 0.290)
Gender	Male	47 (65.3)	5 (71.4)	–	–
	Female	25 (34.7)	2 (28.6)	0.75 (0.10–3.77, *p* = 0.744)	–
ASA	Under III	55 (68.8)	1 (12.5)	–	–
	III and above	25 (31.2)	7 (87.5)	15.40 (2.55–296.00, *p* = 0.013)	14.03 (1.55–127.16, *p* = 0.019)
Time to operation	<6 h	25 (31.6)	1 (12.5)	–	–
	>6 h	54 (68.4)	7 (87.5)	3.24 (0.54–62.28, *p* = 0.283)	2.46 (0.25–24.36, *p* = 0.442)
WHO safer surgery checklist used?	No, not available	18 (22.5)	4 (50.0)	–	–
	No, but available	13 (16.2)	1 (12.5)	0.35 (0.02–2.69, *p* = 0.367)	0.51 (0.04–6.69, *p* = 0.611)
	Yes	49 (61.3)	3 (37.5)	0.28 (0.05–1.36, *p* = 0.112)	0.36 (0.06–2.12, *p* = 0.259)
Antibiotic prophylaxis	No	8 (10.0)	0 (0.0)	–	–
	Yes	72 (90.0)	8 (100.0)	12,849,865.87 (0.00-NA, *p* = 0.994)	–

Univariable and multilevel models for 30-day mortality. Effect estimates are presented as odds ratios (ORs) alongside the corresponding 95% confidence interval

**Table 5:** Model for 30-day surgical site infection

		No	Yes	OR (univariable)	OR (multilevel)
Age (years)	Mean (SD)	22.9 (16)	22.3 (17.8)	1.00 (0.97–1.03, *p* = 0.889)	1.01 (0.97–1.04, *p* = 0.781)
Gender	Male	21 (80.8)	31 (58.5)	–	–
	Female	5 (19.2)	22 (41.5)	2.98 (1.03–10.01, *p* = 0.056)	2.88 (0.86–9.69, *p* = 0.087)
ASA	Under III	22 (75.9)	34 (57.6)	–	–
	III and above	7 (24.1)	25 (42.4)	2.31 (0.88–6.61, *p* = 0.099)	2.27 (0.55–9.33, *p* = 0.256)
Time to operation	<6 h	10 (35.7)	16 (27.1)	–	–
	>6 h	18 (64.3)	43 (72.9)	1.49 (0.56–3.90, *p* = 0.415)	1.46 (0.39–5.48, *p* = 0.573)
WHO safer surgery checklist used?	No, not available	9 (31.0)	13 (22.0)	–	–
	No, but available	5 (17.2)	9 (15.3)	1.25 (0.32–5.22, *p* = 0.755)	0.77 (0.13–4.60, *p* = 0.774)
	Yes	15 (51.7)	37 (62.7)	1.71 (0.59–4.84, *p* = 0.313)	1.99 (0.31–12.96, *p* = 0.471)
Antibiotic prophylaxis	No	3 (10.3)	5 (8.5)	–	–
	Yes	26 (89.7)	54 (91.5)	1.25 (0.24–5.48, *p* = 0.775)	0.29 (0.02–3.90, *p* = 0.350)

Univariable and multilevel models for 30-day mortality. Effect estimates are presented as odds ratios (ORs) alongside the corresponding 95% confidence interval

Following surgery for intestinal typhoid, a high surgical site infection rate was observed at 67.0% (59/88). There was also a high organ space infection rate at 10.2% (9/88). Children had a similar rate of SSI to adults (67.6 vs. 66.7%). An ASA grade of III and above was weakly associated at the univariable level with surgical site infection (OR 2.31, 95% CI 0.88–6.61, *p* = 0.099) and female sex (OR 2.98, 95% CI 1.03–10.01, *p* = 0.056). When explanatory variables were adjusted for in multivariable analysis, there were no associations between entered explanatory factors and SSI.

### Sensitivity analyses

We compared the patient characteristics and the outcomes of both the GlobalSurg 1 and GlobalSurg 2 studies (Tables s1 and s2). The event rates of organ space infection (*p* = 0.062) and mortality (*p* = 0.062) were lower in the GlobalSurg 2 study; however, most other characteristics remained comparable.

## Discussion

This international, multicentre, prospective study has demonstrated substantial post-operative morbidity following surgery for intestinal typhoid. We identified a 30-day post-operative mortality rate of 9.1%, which was higher in children, and a high surgical site infection rate of 67.0%. As typhoid and paratyphoid disproportionately affects patients in LMICs, this high complication rate following surgery has substantial repercussions for both patients and healthcare systems. Concerningly, children appear to have similarly poor outcomes following surgery for typhoid perforation. The incidence of typhoid and paratyphoid is widespread and remains a key public health issues across LMICs [17]. With growing resistance to first-line antimicrobials, surgery for intestinal typhoid is likely to become more common and the healthcare burden of post-operative complications will continue to increase [18]. Surgical site infection, for instance, is not only expensive for healthcare systems, but associated with serious complications and longer lengths of stay [19]. In LMICs, prolonged hospital admissions after surgery for typhoid in patients of working age can result in the absence from work and societal commitments, which subsequently impacts on family units and communities. In children, this can mean time away from education, which is particularly important in the context of wider efforts to reduce inequalities in low- and middle-income countries. Patients and families who have lost earnings and are required to pay for a prolonged treatment are at risk of catastrophic impoverishment with dire long-term consequences [20].

We identified high morbidity rates associated with surgery for typhoid perforation. The reasons for this relate to the disease itself, as well patient and broader health system factors. Morbidity rates may reflect international variation in access to care and to supporting resources to enable clinical staff to deliver safe surgery. Several other reasons for this disparity should also be considered. Firstly, lack of timely access to high-quality medical care may lead to delays in presentation leading to more advanced disease requiring surgical care. Secondly, a lack of capacity in surgery and perioperative care in LMIC settings may compound risks of delay in presentation, leading to preventable SSI and death [21]. Lack of appropriate post-operative care, including intensive care facilities, may contribute to this. Finally, shortages of facilities to perform routine microbiological testing may result in ineffectual prophylactic antibiotic usage where antimicrobial resistance exists.

This study employed a multicentre, prospective approach with standardised outcome assessment and definitions. This contrasts with much of the current literature describing outcomes following surgery for intestinal typhoid, which are retrospective in nature. Our study found markedly higher complication rates compared to the current literature, with a surgical site infection between 20 and 40% higher than those previously reported. This is likely due to the prospective nature of our study and the fact that validated outcome definitions were employed for the diagnosis of SSI, and all collaborators were trained in SSI assessment using online quality assurance modules. Much of the current evidence is also single centre in nature, which may reduce its applicability and generalisability across already diverse LMIC populations. In contrast to this, our study included patients across 15 countries, capturing practice in a variety of settings. One notable aspect of this study is the variety of presentations captured, including a case of gallbladder typhoid requiring cholecystectomy. Gallbladder typhoid has previously been reported and is famously associated with ‘Typhoid Mary’, an American cook who was found to be an asymptomatic carrier of typhoid, with bacteria residing in her gallbladder [22].

Although the collaborative research methodology employed by this study has been characterised elsewhere in detail, this study to our knowledge is the first to use such an approach to study complications of communicable disease. Our study has shown such data can be collected accurately and reliably, demonstrated by validation which has found the data set to be 93.3% accurate. Collaborative working enables additional resources and research infrastructure to be made available in settings where resources may be otherwise limited.

There are several limitations which must be considered when interpreting the results of this study. The sample size is small, although 88 patients undergoing surgery are a large series of intestinal typhoid compared with the existing literature. Secondly, this is a study which pools together data from two of the GlobalSurg studies. A period of 12 months exists between the first and second GlobalSurg study, which may lead to a temporal bias from trends in typhoid incidence. However, in sensitivity analyses comparing both studies limited differences were seen. We did not collect data surrounding the serotype or other specific microbiological parameters of typhoid or paratyphoid as doing so would have been impractical within the confines of this study and limited access to diagnostic resources in included settings. This therefore made it impractical to collect information on antibiotic therapy, including type of antibiotics and administration patterns, as without knowing which bacteria are likely to be present it would not be possible to estimate whether this was appropriately targeted. Finally, as this study was purely observational in nature no causative inference can be drawn between factors associated with outcomes in this study.

Surgery for typhoid is accompanied by substantial morbidity and disproportionately affects a young LMIC population, as reported in the previous studies [4, 21, 23]. Subsequently, funders and policymakers should identify means of reducing the overall burden of morbidity and impact of typhoid upon communities. This should aim to address three key areas: disease prevention, access to prompt surgical care and on improving outcomes. Children are a key group who we have demonstrated to have high mortality rates, and improving outcomes in this group should be a priority. Prevention of typhoid through improved sanitation and vaccination programmes should continue and efforts increased. Improving access to health care, particularly surgical care, at an earlier time in the disease process should be a priority. Future research should focus on identifying durable vaccinations for typhoid, addressing inequalities in access to health care and reducing morbidity rates following surgery for typhoid. Addressing these issues is particularly important, as resistance to antimicrobial agents is increasing. Preventing typhoid will reduce antibiotic usage and preserve current antibiotics for use in severe cases.

## Summary and recommendations

1. Surgery for typhoid is accompanied by substantial morbidity LMIC population as reported in previous studies. Almost half of the included study population were children, who similarly high mortality rates and morbidity rates.
2. Funders and policymakers should identify means of reducing the overall burden of morbidity and impact of typhoid upon communities. This should aim to address three key areas: sanitation, vaccination and access to prompt surgical care.
3. Focus should shift to prevention of typhoid through improved sanitation and vaccination programmes. This approach has had great successes recently, with the eradication of smallpox and polio. Preventing typhoid will reduce the number of patients requiring operation and ensure patients can enjoy fewer absences at work or school. Improving access to health care, particularly surgical care, at an earlier time in the disease process should be a priority. Doing so will enable patients with typhoid to present to health care earlier point in their disease process where antibiotic therapy may be more effective and an operation avoided. Addressing these issues is particularly important, as resistance to antimicrobial agents is increasing. Delivery of non-antibiotic means of preventing typhoid will reduce antibiotic usage and preserve current antibiotics for use in severe cases.
4. Future research should focus on identifying means of reducing morbidity in patients undergoing surgery for typhoid perforation, in particular reducing the incidence of surgical site infection. Further trials should attempt to identify the optimal skin preparation agent, the role of antibiotic prophylaxis and whether the use of laparoscopy may reduce infection rates after surgery for typhoid perforation.

## Electronic supplementary material

Below is the link to the electronic supplementary material.

Supplementary material 1 (DOCX 16 kb)
