# Supplementary material for: Management and Outcomes Following Surgery for Gastrointestinal Typhoid: An International, Prospective, Multicentre Cohort Study
Source: World J Surg. 2018 May 3;42(10):3179–88. doi: 10.1007/s00268-018-4624-8 (PMC6132852; doi:10.1007/s00268-018-4624-8)
Supplement: Supplementary file 1 — Supplementary material 1 (DOCX 16 kb) [file 268_2018_4624_MOESM1_ESM.docx]

# **Supplementary tables**

# Table S1 - Demographic differences GlobalSurg 1 and 2

|  |  | GlobalSurg 1 N = 29 | GlobalSurg 2 N = 59 | P-value |
| --- | --- | --- | --- | --- |
| Age (years) | Mean (SD) | 25.6 (23.1) | 21 (13.3) | 0.926* |
| Gender | Male | 21 (72.4) | 31 (52.5) | 0.053 |
|  | Female | 8 (27.6) | 19 (32.2) |  |
|  | Missing | 0 (0.0) | 9 (15.3) |  |
| ASA | I (normal/healthy) | 10 (34.5) | 18 (30.5) | 0.145 |
|  | II (mild systemic disease) | 6 (20.7) | 20 (33.9) |  |
|  | III (severe systemic disease) | 7 (24.1) | 16 (27.1) |  |
|  | IV (severe systemic disease, constant threat to life) | 4 (13.8) | 3 (5.1) |  |
|  | V (not expected to survive without the operation) | 0 (0.0) | 2 (3.4) |  |
|  | Unknown | 2 (6.9) | 0 (0.0) |  |
| Smoking status | Non-smoker | 22 (75.9) | 52 (88.1) | 0.089 |
|  | Current smoker | 7 (24.1) | 5 (8.5) |  |
|  | Missing | 0 (0.0) | 2 (3.4) |  |
| Diabetes | No | 27 (93.1) | 58 (98.3) | 0.206 |
|  | Yes | 2 (6.9) | 1 (1.7) |  |
| Antibiotic prophylaxis | No | 1 (3.4) | 7 (11.9) | 0.197 |
|  | Yes | 28 (96.6) | 52 (88.1) |  |
| Time to operation | <6 hours | 7 (24.1) | 19 (32.2) | 0.552 |
|  | >6 hours | 22 (75.9) | 39 (66.1) |  |
|  | Missing | 0 (0.0) | 1 (1.7) |  |
| Bowel resection | No | 22 (75.9) | 0 (0.0) | <0.001 |
|  | Yes | 7 (24.1) | 59 (100.0) |  |
| WHO safer surgery checklist used? | No, not available | 12 (41.4) | 10 (16.9) | 0.014 |
|  | No, but available | 6 (20.7) | 8 (13.6) |  |
|  | Yes | 11 (37.9) | 41 (69.5) |  |

#

Numbers are n (%), unless otherwise indicated. All tests are chi-square, except when indicated by *, where a Kruskall-Wallis test has been applied.

# Table S2 - Demographic differences GlobalSurg 1 and 2

|  |  | GlobalSurg 1  N = 29 | GlobalSurg 2  N = 59 | P-value |
| --- | --- | --- | --- | --- |
| Mortality (30 days) | Alive | 24 (82.8) | 56 (94.9) | 0.062 |
|  | Died | 5 (17.2) | 3 (5.1) |  |
| Reintervention (30 days) | No | 23 (79.3) | 46 (78.0) | 0.885 |
|  | Yes | 6 (20.7) | 13 (22.0) |  |
| Surgical site infection (30 days) | No | 12 (41.4) | 17 (28.8) | 0.238 |
|  | Yes | 17 (58.6) | 42 (71.2) |  |
| Organ space infection (30 days) | No | 23 (79.3) | 55 (93.2) | 0.062 |
|  | Yes | 6 (20.7) | 3 (5.1) |  |
|  | Missing | 0 (0.0) | 1 (1.7) |  |

Numbers are n (%), unless otherwise indicated. All tests are chi-square.
